# Targeting Microglia in Neuroinflammation: H3 Receptor Antagonists as a Novel Therapeutic Approach for Alzheimer’s Disease, Parkinson’s Disease, and Autism Spectrum Disorder

**DOI:** 10.3390/ph17070831

**Published:** 2024-06-25

**Authors:** Shilu Deepa Thomas, Sabna Abdalla, Nermin Eissa, Amal Akour, Niraj Kumar Jha, Shreesh Ojha, Bassem Sadek

**Affiliations:** 1 Department of Pharmacology & Therapeutics, College of Medicine and Health Sciences, United Arab Emirates University, Al Ain P.O. Box 15551, United Arab Emirates; 700039712@uaeu.ac.ae (S.D.T.); 202070050@uaeu.ac.ae (S.A.);; 2 Zayed Center for Health Sciences, United Arab Emirates University, Al-Ain P.O. Box 1551, United Arab Emirates; 3 Department of Biomedical Sciences, College of Health Sciences, Abu Dhabi University, Abu Dhabi P.O. Box 59911, United Arab Emirates; 4 Department of Biopharmaceutics and Clinical Pharmacy, School of Pharmacy, The University of Jordan, Amman 11942, Jordan; 5 Centre for Global Health Research, Saveetha Medical College, Saveetha Institute of Medical and Technical Sciences, Saveetha University, Chennai 602105, India; 6 Centre of Research Impact and Outcome, Chitkara University, Rajpura 140401, India; 7 School of Bioengineering & Biosciences, Lovely Professional University, Phagwara 144411, India; 8 Department of Biotechnology, School of Applied & Life Sciences (SALS), Uttaranchal University, Dehradun 248007, India

**Keywords:** microglia, neuroinflammation, cytokines, H3R antagonists, neurogenesis, amyloid beta (Aβ), α-synuclein, Alzheimer’s disease, Parkinson’s disease, autism spectrum disorder

## Abstract

Histamine performs dual roles as an immune regulator and a neurotransmitter in the mammalian brain. The histaminergic system plays a vital role in the regulation of wakefulness, cognition, neuroinflammation, and neurogenesis that are substantially disrupted in various neurodegenerative and neurodevelopmental disorders. Histamine H3 receptor (H3R) antagonists and inverse agonists potentiate the endogenous release of brain histamine and have been shown to enhance cognitive abilities in animal models of several brain disorders. Microglial activation and subsequent neuroinflammation are implicated in impacting embryonic and adult neurogenesis, contributing to the development of Alzheimer’s disease (AD), Parkinson’s disease (PD), and autism spectrum disorder (ASD). Acknowledging the importance of microglia in both neuroinflammation and neurodevelopment, as well as their regulation by histamine, offers an intriguing therapeutic target for these disorders. The inhibition of brain H3Rs has been found to facilitate a shift from a proinflammatory M1 state to an anti-inflammatory M2 state, leading to a reduction in the activity of microglial cells. Also, pharmacological studies have demonstrated that H3R antagonists showed positive effects by reducing the proinflammatory biomarkers, suggesting their potential role in simultaneously modulating crucial brain neurotransmissions and signaling cascades such as the PI3K/AKT/GSK-3β pathway. In this review, we highlight the potential therapeutic role of the H3R antagonists in addressing the pathology and cognitive decline in brain disorders, e.g., AD, PD, and ASD, with an inflammatory component.

## 1. Introduction

Histamine achieves its physiological impact through interactions with four subtypes of G protein-coupled receptors (GPCRs), namely H1, H2, H3, and H4 receptors (H1–4R). Notably, these have served as valuable focal points for drug development. For instance, the H1R, which is primarily targeted by antihistamines such as diphenhydramine and desloratadine, is commonly prescribed to alleviate allergy symptoms. Meanwhile, histamine H2R antagonists like ranitidine are utilized to treat gastric ulcers by mitigating the secretion of gastric acid [1,2]. These classes of drugs have achieved significant global success and widespread utilization. Additionally, the H3R contribute to neurotransmission within the central nervous system, thereby influencing cognitive modulation [3]. The H3R are primarily found in the presynaptic neurons, where they act as auto-receptors modulating the synthesis and release of histamine. Additionally, H3R also act as hetero-receptors, influencing additional neurotransmitters such as GABA, dopamine, glutamate, serotonin, norepinephrine, and acetylcholine [4]. The histamine H4R is expressed in various cell types, including dendritic cells, T cells, basophils, eosinophils, and mast cells. Antagonists targeting the H4R exhibit anti-inflammatory properties and hold promise for treating both inflammation and pruritus [5]. The histaminergic system is pivotal in regulating alertness, cognitive function, inflammatory response in the brain, and neurogenesis [6,7,8].

It is now recognized that both neurodevelopmental and neurodegenerative disorders involve overlapping cellular and molecular mechanisms [9]. Neuroinflammation, synaptic dysfunction, and stem cell depletion are observed in both neurodevelopmental and neurodegenerative disorders [10]. Abnormalities in the histaminergic system are believed to potentially contribute to neurodegenerative disorders, like Alzheimer’s disease (AD) and Parkinson’s disease (PD) [11,12]. AD is an age-related degenerative neurological condition that leads to a gradual and irreversible decline in cognitive function. It leads to cognitive impairment and dementia among the elderly population. AD is classified as a mixed proteinopathy marked by the existence of two primary pathological features: extracellular amyloid beta (Aβ) plaques and intracellular neurofibrillary tau tangles (NFTs) [13]. In specific brain regions, AD also involves glial activation, neuronal loss, synaptic remodeling, and impaired synaptic function [14]. PD results from the gradual and continuous degeneration of dopaminergic neurons in the substantia nigra pars compacta [15]. The symptoms include bradykinesia, rigidity, rest tremor, and impaired gait [16]. Microglia, the primary immune cells residing in the central nervous system (CNS), are thought to be essential contributors in the development of disorders associated with the CNS. Various stimuli can activate microglia, leading to increased neuroinflammation and the progression of brain disorders [17]. Microglia can exhibit different M1/M2 phenotypes depending on the specific microenvironment, and maintaining a balanced ratio between these phenotypes is essential for restoring CNS homeostasis [18]. Microglial activation and proliferation in the brain is a prominent feature of AD and PD [19,20,21]. 

A recent review delved into the correlation between brain development and altered neuroinflammation, highlighting its impact on synaptogenesis and synaptic plasticity. The review put forward the idea that a deficiency in brain histamine might render the developing brain susceptible to inflammatory challenges and neurodevelopmental disorders encompassing Tourette’s syndrome (TS), autism spectrum disorder (ASD), and attention-deficit hyperactivity disorder (ADHD), as indicated by outcomes from both preclinical and clinical studies [22]. ASD is a diverse group of pervasive developmental disorders characterized by deficiencies in social interaction skills, difficulties in communication, and the presence of repetitive behaviors. These traits manifest early in childhood, beginning at birth, and persist throughout adulthood, leading to lifelong disabilities [23]. Increasing evidence suggests a link between alterations in neuroimmunity and the development of ASD and AD. Patients with ASD and AD exhibit similar patterns of cognitive deficits, impaired sociability, and abnormal behavior [24]. The literature increasingly emphasizes important shared features and potential common pathomechanisms in AD, PD, and ASD. Alterations in the key proteins associated with neurodegeneration, such as Aβ, tau, and α-synuclein, are increasingly implicated in the etiopathology of ASD [25].

Exploring antagonists of H3R has been under scrutiny as a therapeutic for various CNS disorders, encompassing AD, epilepsy, narcolepsy, ADHD, and learning deficits induced by fetal alcohol exposure [26,27]. Pitolisant, a H3R antagonist and inverse agonist, has obtained approval for the treatment of narcolepsy in the EU. Preclinical studies suggested the favorable impact of H3R antagonists, such as pitolisant, as they exhibit a positive effect in improving memory impairments, particularly in AD models. Currently, pitolisant is undergoing trials for the treatment of photosensitive epilepsy, as well as in children with ASD, showcasing its progress as a H3R antagonist with promising therapeutic potential [28]. In recent times, considerable research has been conducted on the role of histamine in microglia-mediated inflammation. An emerging therapeutic approach to enhance the treatment of neurocognitive disorders involves the inhibition of excessive microglial activation and the preservation of a balanced microglial phenotypic profile. Studies have reported that H3R antagonists exhibit cognition-enhancing effects, in addition to the suppression of inflammatory responses through inhibiting the activation of glial cells [8,29]. This review offers a comprehensive summary and consolidates the current understanding of experimental and clinical discoveries on the involvement of H3R antagonists in the modulation of microglial functions. Additionally, it explores their potential application in the treatment of neurodegenerative and neurodevelopmental disorders like AD, PD, and ASD. 

## 2. Microglia-Mediated Neuroinflammation 

The primary and principal form of active innate immune defense in the CNS is represented by a distinct population of macrophage-like cells known as microglia [30]. These cells are critical for the balance and stability of the brain’s microenvironment, and their actions are determined by the external signals they receive [31]. Neuroinflammation constitutes an immune response triggered by infections, oxidative stress, irritants, or damage within neuronal tissues. It has been linked to a decline in cognitive function and an elevated risk of dementia [32]. Microglia play a crucial role in detecting and eliminating undesired neuronal connections [33]. This process, known as synaptic pruning, strengthens neural circuits and enhances neuronal network efficiency. The dynamic interaction between neurons and microglia is not only essential for the growth and maturation of the brain but also plays a pivotal role in processes such as synaptic plasticity, contributing significantly to memory and learning [34]. In the resting state, factors such as BDNF released by microglia are essential for maintaining homeostatic synaptic plasticity. These factors are involved in regulating processes like long-term potentiation (LTP) and the excitatory and inhibitory neurotransmission at the synapses [19,35]. Additionally, microglia contribute to critical facets of brain function, such as supporting neuronal survival, promoting neurogenesis, and facilitating oligodendrogenesis [17]. 

Microglia, when exposed to different stimuli such as debris, protein aggregates, and pathogens, undergo phagocytic activation. Termed microglial activation, the process implies a balance between the production of proinflammatory cytokines and the synthesis and release of anti-inflammatory molecules. This equilibrium prevents an exaggerated inflammatory response and microglial activation, safeguarding against potential tissue damage and the interruption of brain homeostasis [36,37]. Microglia undergo divergent differentiation in response to cytokines or lipopolysaccharide (LPS) stimulation. This differentiation leads to the polarization of microglia in two distinct states: classically activated microglia (M1) and alternatively activated microglia (M2). M2 microglia further demonstrate refined phenotypes, such as adopting the type 2 alternative activation (M2b) and acquired deactivated states (M2c) [38]. M1 microglia are distinguished by the release of proinflammatory mediators, contributing to oxidative and nitrosative stress, ultimately leading to tissue damage caused by inflammation. On the other hand, M2 microglia elicit beneficial outcomes through the release of neurotrophic factors and anti-inflammatory cytokines. These factors aid in resolving inflammation and orchestrating neural repair and restoration [17,39]. 

Under normal conditions, microglia keep a dormant neuron-specific monitoring state (M0 phenotype). However, they actively extend processes from their cell bodies, consistently monitoring the brain for potential damage. This ongoing surveillance involves a comprehensive assessment of the brain for any signs of harm at regular intervals. M1 microglia, recognized as proinflammatory cells, swiftly respond to appropriate stimuli and act as the initial line of defense for the neuroimmune system, typically appearing within hours of stimulation. Using immune receptors like Toll-like receptors, microglia recognize harmful stimuli. Upon activation, M1 microglia release high levels of proinflammatory cytokines [40]. M1 microglia, expressing NADPH oxidase and iNOS, produce ROS and nitric oxide, contributing to neurological damage. M1 microglia express various molecules like MHC-II; integrins (CD11b and CD11c); and various costimulatory molecules (CD36, CD45, and CD47) and Fc receptors. In contrast, M2 microglia with anti-inflammatory functions release neuroprotective factors. They release growth factors (FGF, CSF-1, NGF, BDNF, neurotrophins, and GDNF), promoting inflammation resolution, tissue repair, and neuron survival [41]. In response to pathological injury, microglial cells swiftly undergo activation and polarization, exhibiting various behaviors such as phagocytosis, chemotaxis, and cytokine secretion. The nature of their response depends on the severity of the injury. Mild injuries lead to the activation of microglial cells into the M2 state, marked by distal branches, small cell body-like changes, and the secretion of anti-inflammatory factors contributing to tissue repair and neuroprotection [42]. However, severe or prolonged tissue damage triggers the complete activation of microglial cells into the M1 state characterized by amoeboid changes and thick protrusions [43]. 

Upon recurrent stimuli, such as the accumulation of Aβ in the initial phases of AD, activated microglia undergo a shift towards a less defensive and more neurotoxic state known as “primed” microglia [44]. This shift is characterized by an increased sensitivity to proinflammatory stimuli, a phenomenon known as microglia priming. Aging microglia also exhibit increased sensitivity to proinflammatory stimuli [36]. The heightened response of primed microglia involves the release of proinflammatory cytokines. In the natural aging process, microglia show a tendency towards adopting the conventional M1 phenotype, resulting in an increased baseline release of proinflammatory cytokines. These cytokines have a negative impact on neurogenesis in the hippocampus. This persistent activation, release of proinflammatory cytokines, and a reduction in phagocytic activity ultimately result in reduced synaptic plasticity and the impairment of learning and memory [45]. Hence, in pathological circumstances, microglia undergo a shift from their typical “homeostatic state” to an activated “disease-associated state”. With the progression of the disease, microglia may gradually lose their ability to regulate effectively and transition into a “neurodegenerative disease state”. This transition renders them dysfunctional and potentially harmful to the CNS [46]. 

Astrocytes are another group of specialized glial cells with diverse functions, essential for brain homeostasis, neuronal development, synaptic connectivity, and the support and protection of neurons in the CNS [47]. Astrocytes form intricate networks with neurons and other cells. During early neurodevelopment, astrocytes promote the formation, migration, and neuronal cells connectivity, promoting the formation of synapses and establishment of neuronal circuits [48]. They establish tripartite structures with neurons, contributing to synaptic communication. Astrocytes have the ability to detect and react to alterations in their nearby environment, including neurotransmitters, to modulate neuronal signaling and provide protection to neurons against oxidative damage and injuries [22,49,50]. The activation of astrocytes induces the generation of proinflammatory cytokines, chemokines, and ROS. This activation can also stimulate microglial activity. Consequently, these processes contribute to heightened excitotoxicity, apoptosis, and neurodegeneration [22]. 

Microglia and astrocytes can polarize into two states: proinflammatory (M1 and A1) and anti-inflammatory (M2 and A2). Microglia are a more attractive therapeutic target than astrocytes in inflammatory neurological diseases, as they are the primary responders to damage, thereby initiating and sustaining the immune response [51]. The activation of pattern recognition receptors (PRRs) by pathogen- or damage-associated molecular patterns (PAMPs/DAMPs) triggers the M1 phenotype in microglia. By targeting microglia, it may be possible to reduce the harmful inflammatory response and protect neurons from secondary damage. Microglia express a variety of receptors, such as TLRs, whereas astrocytes primarily express TLR3, with a limited expression of TLR1, TLR4, TLR5, and TLR9 and no expression of TLR2, TLR6, TLR7, TLR8, and TLR10. The limited expression of TLRs in astrocytes also suggests their reduced capacity to respond directly to various pathogens and instead depend on microglia to detect pathogens and communicate with astrocytes to activate them [52]. In the CNS, TLRs primarily recognize infectious pathogens or endogenous ligands released during injury and stress. The degree of astrocytic responses to TLR ligands, compared to microglia, remains debatable. Astrocytic responses to TLR3 and TLR4 activation are thought to be entirely dependent on microglia. Specifically, TLR4 activation by LPS shows that microglia directly initiate or facilitate astrocytic responses through soluble mediators, highlighting the critical role of microglia–astrocyte communication in CNS injury or inflammation [53]. TNF-α is the most abundant proinflammatory factor released by microglia when activated by TLR4 agonists. Studies show that astrocyte cultures alone release very low levels of inflammatory cytokines, nitric oxide (NO), and ROS in response to LPS stimulation. These findings suggest that astrocytic immune responses under inflammatory conditions rely on microglia, as astrocytes alone do not produce sufficient levels of proinflammatory factors like TNF-α and NO after LPS stimulation [53].

Emerging evidence indicates that activated microglial cells play a crucial role in modulating astrocytosis and determining astrocyte’s fate under various pathological conditions [54]. Astrocyte activation is often secondary to microglial activation, making microglia a more direct target for modulating the primary immune response in the CNS. Microglia can enhance the inflammatory activation of astrocytes by increasing the expression of cytokines and chemokines, particularly through the activation of NF-κB signaling. Reactive microglia produce cytokines that induce the conversion of astrocytes to the neurotoxic A1 phenotype. Once A1 astrocytes are induced, they lose their crucial functions, such as supporting synaptic pruning and neuronal survival, instead promoting neuroinflammation, contributing to the progression of neurodegenerative diseases [50,55]. The release of proinflammatory cytokines also inhibits the expression of Cx-43, a key protein in astrocyte gap junctions. This inhibition hampers astrocytic communication, which may further prevent their neuroprotective functions [56]. AHR signaling in microglia controls the expression of proinflammatory genes (Ccl2, IL-1b, and Nos2) in astrocytes by modulating the microglial production of vascular endothelial growth factor (VEGF)-B and transforming growth factor (TGF)-α. Microglial VEGF-B enhances NF-κB translocation in astrocytes through FLT-1 signaling, promoting their pathogenic activity during EAE, while TGF-α interacts with the ErbB1 receptor in astrocytes to mitigate EAE progression and encourage the production of neuroprotective factors [57]. Microglia also regulate astrocyte functions in neuroinflammation through mechanisms such as stromal cell-derived factor (SDF)1α signaling via C-X-C chemokine receptor (CXCR)4, which drives astrocyte glutamate release. Microglial TNF-α enhances this glutamate release, contributing to neuron excitotoxicity. By modulating microglial activity, it is possible to indirectly regulate astrocytic responses and CNS inflammation. 

Traditionally, researchers have assessed microglial function using morphology and immunochemical markers. The M1–M2 paradigm simplifies microglial activation investigation. However, accumulating evidence challenges this model, suggesting microglia do not strictly adhere to the M1–M2 dichotomy. Transcriptome studies reveal diverse activation patterns in microglia, challenging the notion that M1 and M2 represent distinct cell subtypes. Instead, a spectrum of intermediate phenotypes exists, rendering the current paradigm inadequate for describing in vivo microglial activation. Microglia can transition between phenotypes in different CNS environments, ensuring their protective role by adapting their phenotype as needed. Numerous microglia phenotypes are currently recognized, each characterized by a variety of morphological, molecular, metabolic, and functional traits [37]. The prevailing consensus is that these distinct phenotypes correspond to a range of functions performed by microglia [43]. Despite limitations, the classification is extensively used to illustrate that microglia may exhibit either protective (M2) or detrimental (M1) characteristics dependent on the circumstances [58,59]. This review primarily focusses on the binary categorization of microglia to examine the microglial transition from the M1 to M2 phenotype in AD, PD, and ASD and the role of H3R antagonists as a therapeutic strategy.

### 2.1. Neuroinflammation in Neurodegenerative and Neurodevelopmental Disorders

#### 2.1.1. Neuroinflammation in Alzheimer’s Disease

Neuroinflammation is frequently observed in AD. Microglia are recognized to have significant involvement in regulating this inflammation within the brain [60]. Pascoal et al. (2021) led positron emission tomography imaging studies on individuals with AD, offering evidence that microglial activation in the brain is not solely a consequence of the progression of the disease. Instead, their findings suggest that microglial activation is a crucial upstream mechanism required for the development of the disease [61]. By detecting Aβ aggregates or damage- or pathogen-associated molecular patterns (DAMPs or PAMPs), cells can potentially cause inflammation in the CNS. Numerous pattern recognition receptors (PRRs) are found in cells’ cytoplasm or on their surface, which are accountable for identifying PAMPs and DAMPs [21]. This interaction initiates inflammatory signaling cascades and a range of immune reactions. There are five major PRR families, including the TLR, RLR, NLR, CLR, and ALR. Through this PAMP/DAMP-mediated signaling response, a cascade is initiated that leads to the release of inflammatory cytokines and chemokines, prompting cell death, as well as the elimination of infected cells [62]. 

**Figure 1:**
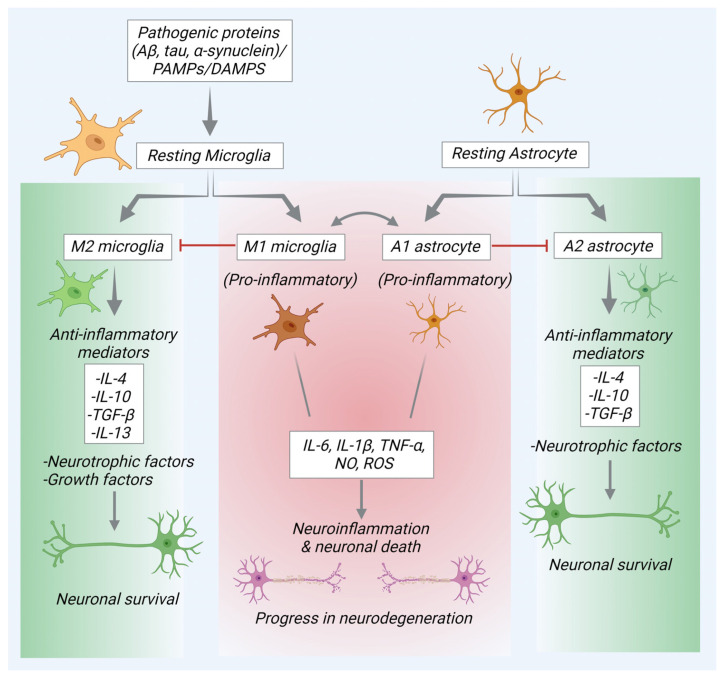
Potential relationship between neurodegenerative diseases and glial cells. The aggregated proteins like amyloid-β, tau, and α-synuclein induce changes in microglia and astrocytes, causing them to adopt proinflammatory phenotypes. Activated microglia are often classified as M1 or M2 phenotypes. Resting microglia polarize to the M1 phenotype and produce proinflammatory substances such as TNFα, IL-1, IL-6, IL-12, NO, and ROS. Activated astrocytes are usually divided into the A1 and A2 phenotypes. The M1 microglia’s production of the proinflammatory cytokines causes the A1 neurotoxic phenotype and encourages the secretion of TNF α, IL-1, and IL-6. The proinflammatory microglia/astrocytes release factors that can disrupt synaptic function and cause neuronal cell damage. These disruptions contribute to the progression of neurodegenerative diseases.

In AD, neuroinflammation in the brain manifests through the activation of microglia and astrocytes and increased levels of proinflammatory cytokines and chemokines. These changes serve as distinct markers of neuroinflammation in AD patients [59,63]. Impairment in the effective clearance of Aβ proteins, either due to neuronal damage or excessive production, disrupts microglial activity. This disruption results in the buildup of Aβ proteins, resulting in plaque formation in the brain. Microglia possess various receptors, such as TLR4 and TLR6, CD36, and NLRP3 domains, that bind to Aβ. These receptors are triggered by DAMPs, including adenosine triphosphate. The binding of Aβ to CD36, TLR4, or TLR6 receptors results in the activation of microglia and subsequent secretion of inflammatory cytokines and chemokines. During microglia activation, key cytokines released include TNF-α and IL-1β. TNF-α binds to its receptor on the cell membrane, initiating apoptosis by means of the extrinsic pathway [64]. Reactive astrocytes significantly contribute to the neuroinflammatory processes linked to AD. Reactive astrocytes that are recruited to Aβ plaques secrete cytokines such as IL-1β and TNF-α. These are potent mediators of inflammation. IL-1β and TNF-α can promote the activation of microglia and stimulate the release of additional proinflammatory molecules, creating a cycle of neuroinflammation [65,66] (Figure 1). In addition to eliminating amyloid oligomers and fibrils, microglia may establish a physical barrier surrounding plaques and fibrils, thus impeding their dissemination and mitigating their toxic effects [67]. The release of certain proteases, that cause the breakdown of amyloid also influence the elimination of amyloid plaques [34,68]. 

However, the persistent activation of microglia by amyloid plaques can be detrimental, leading to chronic inflammation and excessive accumulation of amyloid plaques, thereby accelerating neurodegeneration [68]. During the pathogenesis of AD, there is a heightened production of proinflammatory cytokines and other damaging components [69]. Microglial activation is seen at the pre-plaque stage in preclinical models of AD, suggesting that neuroinflammation occurs early in AD progression [59]. This activation triggers the production of proinflammatory mediators, leading to the overexpression of apoptotic proteins by disrupting the PI3K-AKT-GSK-3β pathway [70]. This signaling pathway governs diverse activities, encompassing cell division, differentiation, cell survival, motility, synaptic plasticity, and memory. GSK-3β (pGSK-3β) activation also promotes apoptosis in various conditions, whereas its inhibition supports cell survival [71]. GSK3β participates in a series of events that include the hyperphosphorylation of tau protein, Aβ production, and local inflammatory responses, all contributing to the initiation and advancement of AD [72]. Throughout adulthood and the aging process, there is the persistent activation of microglia, which contributes to a substantial decrease in neurogenesis. In animal models, this is evidenced by a diminished rate of cell proliferation. This diminished neurogenesis has implications for hippocampal function, resulting in reduced learning and memory capabilities and contributing to cognitive degeneration in aging individuals [73]. Table 1 lists the drugs under investigation in clinical trials targeting neuroinflammation for beneficial effects in AD.

#### 2.1.2. Neuroinflammation in Parkinson’s Disease 

PD involves the gradual degeneration of dopaminergic neurons within the substantia nigra, leading to reduced dopamine release into the striatum. This neurodegenerative process gives rise to movement disorders such as tremors, stiffness, and bradykinesia, as well as cognitive impairments like dementia. Additionally, PD manifests with mental symptoms such as depression and anxiety, along with disruptions in sleep patterns and bowel functions [74,75]. Elevated levels of innate immune system mediators, including chemokines and cytokines released by microglia, are present in the brains of individuals diagnosed with PD. TNF-α, IL-1, and IL-6 have specifically been proposed as potential biomarkers for PD [76]. Elevated levels of TNF-α in the bloodstream were demonstrated in patients with PD experiencing more pronounced cognitive impairment, depression, sleep disturbances, and fatigue [76,77,78]. Several research investigations have shown that aggregated α-synuclein released into the extracellular space from degenerating or deceased dopaminergic neurons can directly stimulate microglial activation toward the M1 phenotype. This activation involves the activation of NADPH oxidase, leading to increased release of ROS and proinflammatory mediators [15,18]. 

Based on the findings from both preclinical and clinical research, the initial microglial response contributes to the improved survival of neurons in addition to the preservation of injured dopaminergic neurons in context of PD [79]. The deregulated release of proinflammatory cytokines, however, has been related to a prolonged hyperactivation of microglia, making them contributors to toxic and neurodegenerative processes in PD [80]. In fact, the postmortem analysis revealed higher amounts of the TNF-α, interleukin-1 (IL-1), IL-2, IL-4, and IL-6, as well as TGF-β1 and basic fibroblast growth factor (bFGF), in PD patients [81]. Similar results, including the detection of IL-1, IL-2, IL-4, TGF-β1, TGF-β2, and TGF-β3, have been observed in the cerebrospinal fluid of subjects. Such cytokine production creates a distinctive network in the brain that affects typical glial and neural function [82]. PD’s ongoing neurodegeneration has been linked to the prolonged microglial neuroinflammatory response, their pathological interactions with neighboring resident glial cells like astrocytes, and the infiltration of immune cells from the periphery, such as lymphocytes and macrophages [83]. Reactive astrocytes were also identified in the substantia nigra pars compacta of individuals with PD. The malfunction of astrocytes plays a role in the neurodegeneration of dopaminergic neurons in PD [84]. The early recognition of neuroinflammation in PD is of paramount importance so as to understand the pathways of disease progression but also for discovering novel targeted opportunities to modulate or obstruct the neuroinflammatory process. Table 2 enlists the drugs under investigation in clinical trials targeting neuroinflammation for the treatment of PD. 

#### 2.1.3. Neuroinflammation in ASD 

Many reviews have focused on the role of microglia and neuroinflammation in ASD [85,86,87]. The etiology of ASD involves the downregulation of genes associated with synaptic function and upregulation of immune-related genes. Postmortem studies in brains of ASD patients show changes in microglia density. Individuals with ASD exhibit microglial activation and neuroinflammation [36]. Various environmental factors play a role in the development of ASD, many of which impact the immune response during prenatal or early postnatal development [88]. The brain regions implicated in autism-related behaviors, particularly limited social interaction and repetitive behaviors, include the hippocampus and cerebellum. Studies suggest that changes in social behavior in adult mice may be attributed to cerebellar inflammation, given its role in cognitive functions [89,90,91]. The activation of astrocytes and microglia in the cortex and cerebellum leads to the increased expression of IL-6, TNF-α, and MCP-1 in different brain regions of individuals with autism [92,93,94]. Hence, the regulation of microglial activation and the suppression of cytokine and free radical production might be a therapeutic approach for treating ASD. Table 3 enlists drugs currently undergoing clinical trials targeting neuroinflammation for the treatment of ASD.

## 3. The Role of H3R Modulators in Neurodegenerative and Neurodevelopmental Disorders

### 3.1. Pharmacology and Signaling of Histamine H3Rs

**Figure 2:**
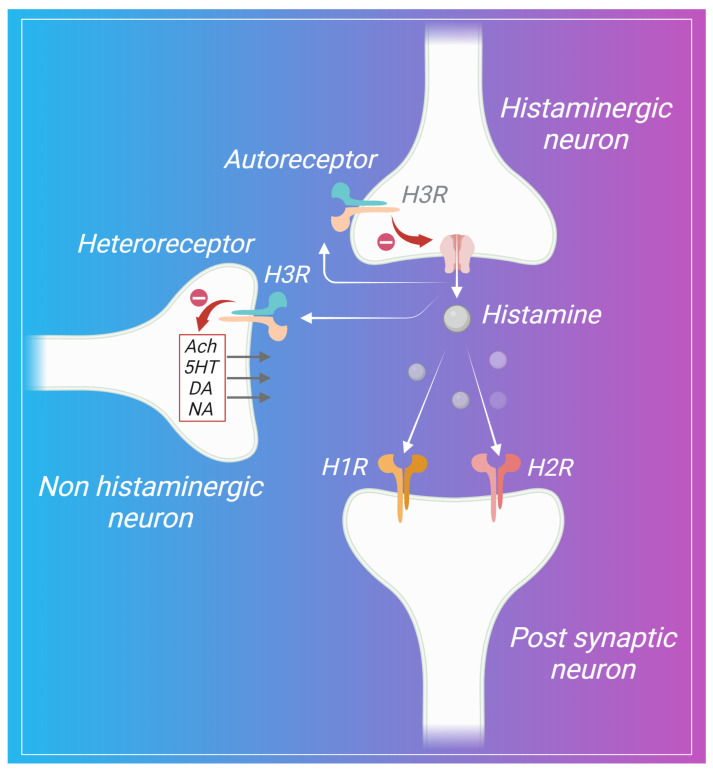
H3 receptors functioning as auto- and heteroreceptors.

Located within the hypothalamus in the tuberomammillary nucleus (TMN), histaminergic neurons serve as the principal source of histamine synthesis in the brain [96]. The intricate mechanisms underlying the synthesis and multifaceted roles of histamine have been comprehensively explored in several notable reviews [97,98,99]. H3R are a type of GPCR that were initially discovered on presynaptic histaminergic neurons, where they function as auto-receptors and limit histamine release in the brain [100]. In addition to their role as auto-receptors, H3R have been observed to regulate the release of various neurotransmitters crucial for cognition, including dopamine, serotonin, and acetylcholine, on non-histaminergic neurons, functioning as heteroreceptors [101,102,103,104,105]. Figure 2 depicts a schematic representation of H3R functioning as auto- and heteroreceptors to modulate histamine and diverse neurotransmitters in the brain. 

**Figure 3:**
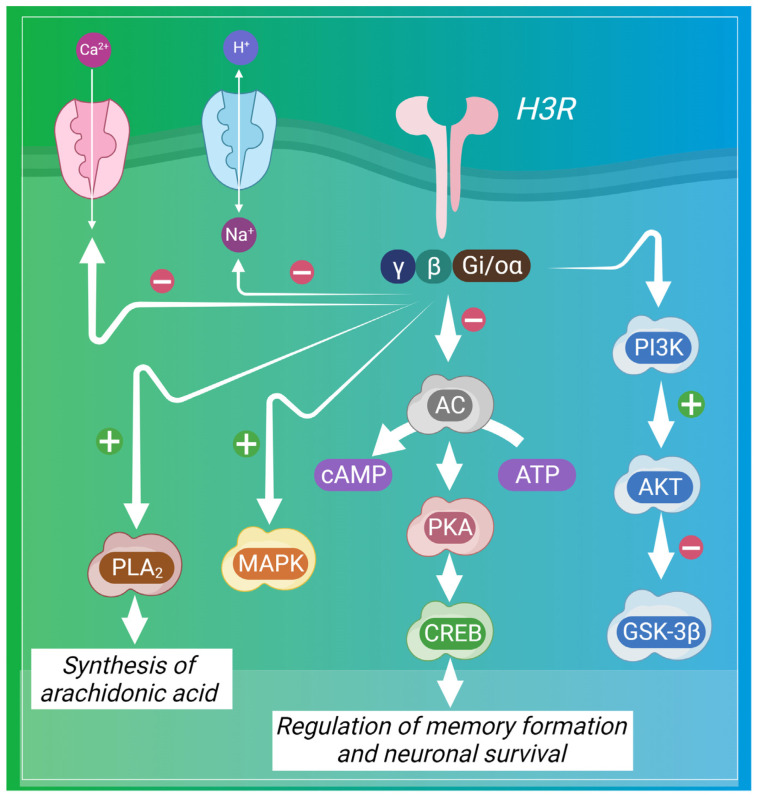
Signaling pathways associated with the histamine H3 receptor. The activation of H3R leads to the activation of Gα_i/o_ proteins and subsequent inhibition of adenylyl cyclase (AC), resulting in the suppression of the cAMP/PKA cascade. Consequently, there is a downregulation of the pro-survival transcription factor CREB. H3 receptor stimulation also activates the MAPK and PI3K pathways. The activation of the PI3K enzyme subsequently activates AKT, leading to the inactivation of the proapoptotic protein GSK-3β through phosphorylation. H3 receptor activation also increases the action of enzyme PLA2, leading to the release of arachidonic acid. H3R also play a role in inhibiting the Na^+^/H^+^ exchanger (NHE), responsible for buffering intraneuronal pH, and reducing the intracellular levels of Ca^2+^.

H3R activation, leads to inhibition of adenylate cyclase, resulting in a decline in the cyclic AMP (cAMP) levels and suppression of the downstream signaling pathways, like the activation of protein kinase A (PKA) and transcription of genes induced by cAMP-responsive element binding protein (CREB). The Gα_i/o_ proteins, which, coupled with H3R, play a role in this inhibition. Additionally, the Gβγ complexes of Gα_i/o_ proteins hinder the opening of voltage-activated calcium channels, leading to a decrease in neurotransmitter release. H3R is also known to form receptor heterodimers with other receptors, such as dopamine D1 and D2 receptors. The activation of H3 receptors triggers various effector proteins such as PI3K, MAPKs, and PLA2. These pathways lead to the phosphorylation of protein kinase B (PKB) by PI3K and activation of extracellular signal-regulated kinases (ERKs) by MAPK. The activation of H3R also inhibits glycogen synthase kinase-3β (GSK-3β) through PKB. Additionally, H3R activation results in the inhibition of the Na^+^/H^+^ exchanger’s activity [4,106]. Figure 3 depicts the diverse signaling pathways mediated by H3R in the CNS. 

### 3.2. Role of Histamine in Microglial Activation

Histamine plays a dual function within the brain, serving as both a neurotransmitter and a modulator of the innate immune system. This dual functionality contributes to its influence on inflammatory reactions in the brain [6,107]. All four subsets of histamine receptors are expressed by microglia, impacting their behavior differently and play a regulatory role in various microglial functions, such as chemotaxis, migration, cytokine secretion, and autophagy [108]. Astrocytes also modulate neuroprotective responses through the expression of H1R, H2R, and H3R. In addition, histamine inhibits the generation of IL-1β and TNF-α while promoting the production of GDNF by astrocytes, thereby exerting neuroprotective effects [109]. Frick et al. (2016) conducted in vivo investigations to examine the impact of histamine on microglial activation. Using immunohistochemistry, the researchers examined the impact of the deficiency of histamine in histidine decarboxylase (Hdc) KO mouse, as well as histamine receptor stimulation in wild-type mice. The study revealed that histamine regulates microglia through H4R [11]. In Hdc KO mice with chronic histamine deficiency, microglia exhibited decreased ramifications and reduced expression of insulin-like growth factor-1, indicating a possible disruption in the histaminergic regulation of microglia. Interestingly, histamine has been described to exhibit both positive and detrimental effects on microglial activity [110]. Ferreira et al. (2012) showed that histamine triggers primary microglial cell motility by activating H4R, dependent upon the presence of α5β1 integrin expression. This process involves the p38 MAPK and AKT signaling pathways. These findings suggest that histamine functions as an inflammatory mediator with harmful effects on the brain. However, histamine reduced the production of IL-1β in response to LPS and diminished the microglial motility, as well as their phagocytic and ROS production capabilities, demonstrating the anti-inflammatory action of histamine as a positive effect on microglial function [111]. Several other studies have confirmed that histamine activates H1- and H4Rs to cause microglia cells to produce proinflammatory cytokines, indicating the adverse impact of histamine on microglial functions [110,112,113]. The subsequent activation through H1- and H4Rs could also be partially hindered by antagonizing both receptor subtypes. 

Recent research has provided additional evidence in support that histamine can reduce the proinflammatory phenotype of microglia, known as SOD1-G93A. This study indicated that histamine’s beneficial effects were specifically observed in the presence of inflammatory SOD1-G93A microglia, while it had a proinflammatory effect on non-transgenic cells [114]. Histamine triggered a decrease in NF-κB and NADPH oxidase 2 (NOX2). The outcomes of the study highlight a distinct function of histamine under normal physiological conditions compared to its role during an inflammatory response. Another study additionally showcased the dual nature of histamine in regulating microglial responses. In vitro experiments were conducted to assess the impact of histamine on microglial phagocytosis and ROS production in the presence of an LPS-induced inflammatory stimulus. Interestingly, both histamine and LPS independently increased FcγR-microglial phagocytosis. However, when exposed simultaneously to histamine and LPS, the increase in microglial phagocytosis induced by each compound individually was inhibited. Moreover, while histamine and LPS separately elevated the microglial ROS levels compared to the control, their combined exposure significantly attenuated these individual effects. It implies that, while histamine can induce a detrimental, proinflammatory phenotype in microglia on its own, it can have the opposite effect when faced with an inflammatory challenge [2]. This discovery opens new possibilities for utilizing histamine therapeutically to specifically improve the processes associated with inflammation in disorders characterized by microglia-induced inflammation. Another study demonstrated that inflammatory stimuli in the periphery, like LPS, can instigate immune memory within the brain. This phenomenon is predominantly facilitated by microglia and can result in either heightened immune response (training) or suppressed immune response (tolerance). This immune memory may play a role in modulating various neurological pathologies and shed light on the dual role of histamine [115]. 

Saika et al. (2020) investigated the intriguing possibility of using chemo-genetic techniques to modulate microglial function [116]. Astrocytic and microglial GPCRs play a crucial role in integrating extracellular signals by regulating intracellular Ca^2+^ signaling and modulating the cAMP levels, as well as the release of various chemo-active substances. Aberrant glial Ca^2+^ signaling and the altered release of chemo-active substances are common in CNS disorders. Targeting microglial and astrocytic GPCRs may offer insights into various pathophysiological conditions and potential therapeutic approaches. Designer receptors exclusively activated by designer drugs (DREADDs) serve as valuable tools for studying GPCR-mediated signaling in astrocytes and microglia. A review by Bossuyt et al. (2023) highlighted the recent findings on the DREADD-induced modulation of astrocytes and microglia [117]. Gi-DREADD activation in microglia has been found to attenuate proinflammatory signaling mediators by inhibiting Ca^2+^ levels and downregulating the transcription factor IRF8 and reducing IL-1β. Gs-coupled signaling, specially β_2_-AR receptor activation, has been found to reduce microglial activation and promote the anti-inflammatory phenotype through the cAMP/PKA/CREB, p38 MAPK and PI3K pathways [118,119,120,121]. Gi-DREADD activation in hippocampal astrocytes further reduced the LPS-induced upregulation of inflammatory markers and alleviated cognitive impairment. In medial basal hypothalamus astrocytes, Gi-DREADD activation decreased IL-1β, TNF-α, CCL2, and CCL5. This modulation of proinflammatory cytokines by DREADDs holds considerable promise for intervening in the neuroinflammation component of AD, PD, epilepsy, multiple sclerosis, and amyotrophic lateral sclerosis [117].

### 3.3. Role of H3R in Microglia Activation and Neuroinflammation

Microglia expresses H3R, and activation of this receptor decreased both forskolin-induced cAMP accumulation and ATP-induced Ca^2+^ transients. Iida and colleagues found that, in mouse primary microglia cultures, H3R activation suppressed essential microglial functions such as chemotaxis, phagocytosis, and the production of proinflammatory mediators [122]. However, in subsequent investigations utilizing ex vivo mouse hippocampal organotypic slices and in vivo models, the authors demonstrated the inhibitory effect of the H3R inverse agonist JNJ10181457 on microglial phagocytosis and cytokine expression. JNJ10181457 inhibited microglial chemotaxis in hippocampal slices and reduced the phagocytosis of dead cells. In vivo phagocytosis was also inhibited by JNJ10181457. It also suppressed LPS-induced expression of IL-1β, IL-6, and TNFα in microglia, further indicating the inhibition of microglial activation. Additionally, it showed efficacy in ameliorating depression-like behaviors induced by LPS [123]. Furthermore, similar effects were also observed with thioperamide, a H3R antagonist, as it alleviated neuroinflammation in LPS-treated BV2 microglial cells. Under LPS-induced neuroinflammatory conditions, inhibiting H3R with thioperamide significantly reduced microglial activation. Additionally, thioperamide decreased the transcription and expression of proinflammatory cytokines [124]. These findings highlight the significant influence of the local environment on microglial activity, suggesting that varying extracellular microenvironments could lead to different roles for H3R in microglial functions both in vitro and in vivo. 

### 3.4. Involvement of Brain H3R in Neurodegenerative and Neurodevelopmental Disorders 

#### 3.4.1. Alzheimer’s Disease 

In the development of AD, neuroinflammation has been identified as a pivotal factor. Among the innate immune cells, microglia emerge as central participants in this inflammatory process. Activated microglia exhibit a range of phenotypes and engage in intricate interactions with Aβ and tau species, as well as neuronal circuits [59]. In AD, microglia respond to Aβ by producing various proinflammatory mediators. This activation of microglia subsequently triggers the activation of astrocytes. The activated astrocytes become involved in the inflammatory process alongside microglia, as described by Osborn et al. in 2016 [125]. Activation of both microglia and astrocytes, particularly the A1-type astrocytes, can contribute to progression of the disease by releasing proinflammatory cytokines that can induce neuronal damage, leading to cognitive deficits [55,126]. 

The disruption of hippocampal neurogenesis is also a key factor in the cognitive deterioration seen in AD. Persistent neuroinflammation is identified to have detrimental effects on hippocampal neurogenesis [127,128]. Reduced neurogenesis in the subventricular zone (SVZ) and subgranular zone (SGZ) has been convincingly linked to the neuroinflammatory states. This correlation may offer insights into the impaired formation of short-term memories observed in individuals with AD. Histamine has been demonstrated to boost neurogenesis and facilitate neuronal differentiation and the dendritic arbor complexity. Blocking the H3R has been found to promote neurogenesis in conditions such as traumatic brain injury and aging. Furthermore, it aids in the migration of neural stem cells (NSCs) [6,129,130]. Investigations have indicated the involvement of microglia in neurogenesis [39,131]. M2 microglia polarization driven by IL-4 in the hippocampus has been shown to stimulate BDNF-dependent neurogenesis. On the other hand, mediators associated with M1 microglia, such as IL-6, IL-1β, and TNF-α, are identified to have an adverse effect on hippocampal neurogenesis [132,133]. They achieve this by diminishing the proliferation and viability of newly formed cells. The agents that can induce M2 polarization or prevent M1 polarization can be of potential therapeutic value in AD. 

Guilloux and colleagues demonstrated that the chronic administration of S38093, a H3R antagonist/inverse agonist, enhances hippocampal neurogenesis in young adult and aged mice, as well as in a transgenic model of AD. Furthermore, improvement was also observed in the performance of aged mice in a context discrimination test [134]. Selective inactivation of H3Rs has been found to boost the process of neurogenesis in the hippocampal subgranular zone, a critical region for cognitive functions and vulnerable to AD [96]. Accordingly, S38093 administration resulted in increased neurogenesis in the hippocampal region in adult 129/SvEvTac mice and aged C57Bl/6JRj mice, as well as in a rodent model of AD (APPSWE). These effects were achieved through the stimulation of BDNF-IX, BDNF-IV, and BDNF-I transcripts, as well as increased expression of vascular endothelial growth factor in aged mice [134]. 

Deficits in the CREB signaling pathway are seen in AD pathology [135]. Research suggests that the activation of CREB leads to a decreased expression of NF-κB and proinflammatory cytokines [136]. Activation of the CREB pathway has also been shown to promote microglial polarization towards a M2 phenotype, characterized by anti-inflammatory properties [137]. The activation of downstream signaling pathways of H3R, such as CREB, has been linked to ameliorating cognitive impairments and reducing Aβ pathology in AD [138]. Interestingly, this has also been shown to suppress inflammatory responses by inhibiting the activation of glial cells [8]. H3R antagonists exhibit neuroprotective effects through the cAMP/CREB and PI3K/AKT/GSK3β signaling cascades [106,139]. Wang et al. (2019) investigated a novel compound called LC1405, a H3R antagonist in mitigating cognitive deficits induced by Aβ. The outcomes of the research demonstrated the effectiveness of LC1405 in slowing down the disease advancement in a mouse model of AD (APP/PS1). It enhanced memory and learning abilities, preventing neurodegeneration and structural abnormalities. Additionally, it alleviated cholinergic dysfunction. LC1405 administration resulted in the upregulation of acetylcholine and histamine, activating neuroprotective cAMP/CREB and AKT/GSK3β cellular signaling pathways mediated through H3R [140]. 

**Figure 4:**
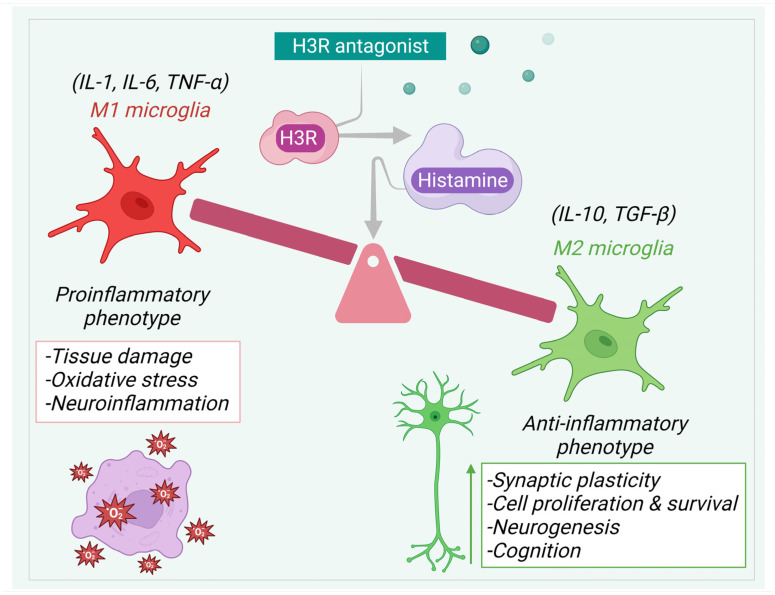
Schematic representation of the neuroprotective action exhibited by H3 receptor antagonists. The inhibition of H3R results in decreased activity of microglial cells, which encourages a shift from the proinflammatory M1 state to an anti-inflammatory M2 state. This transition alleviates neuroinflammation and supports hippocampal neurogenesis, ultimately enhancing cognitive function.

In a study involving APP/PS1 mice, H3 receptor antagonist thioperamide reduced inflammation within the hippocampus and cerebral cortex, with decreased reactivity of the astroglial and microglial cells. Inhibition by thioperamide transformed the astrocytes from a reactive A1 state to a protective A2 state and suppressed the expression of phosphorylated p-P65 NF-κB. These effects were mediated through the cAMP/CREB pathway. Thioperamide exhibited the ability to block gliosis and the release of proinflammatory cytokines, but H89, a CREB signaling inhibitor, eliminated these effects. In addition, thioperamide reduced cognitive decline and Aβ-deposition. However, these effects were reversed upon treatment with H89. Hence, H3R antagonists have anti-inflammatory effects in the brain by modulating astrocyte reactivity and reducing inflammation-associated markers [8]. Thioperamide was also found to reduce microglial activity, suppress inflammation, and promote neurogenesis in the LPS-induced model of neuroinflammation [124]. Furthermore, thioperamide alleviated neuroinflammation in BV2 microglial cells in vitro. Through the activation of the PKA/CREB pathway, thioperamide facilitated the transition of microglia from a proinflammatory M1 phenotype to an anti-inflammatory M2 phenotype. Simultaneously, it inhibited the NF-κB signaling pathway. Thioperamide’s activation of CREB fostered the interaction between CREB and CREB binding protein (CBP), resulting in the increased secretion of anti-inflammatory cytokines (IL-4 and IL-10) and BDNF. Conversely, it reduced the interaction between NF-κB and CBP, resulting in reduced levels of proinflammatory cytokines. Figure 4 illustrates the neuroprotective action exhibited by H3R antagonists.

The significant resemblance between H3R and H4R leads to notable similarities in ligand affinities, enabling the concurrent activation of both receptors. Dual-acting ligands targeting H3R and H4R hold promise for therapeutic applications across various pathological conditions, including neuropathic pain, cancer, PD, and inflammatory disorders [5]. The manipulation of histaminergic receptors using a pharmacological agent capable of selectively inhibiting H3Rs while stimulating H4Rs has been shown to replicate neuroprotective effects in a mouse model of AD. Clobenpropit, a H3R inverse antagonist and partial H4R agonist, demonstrated a protective effect against amyloid peptide-induced neuronal damage in a rat model of AD [141]. A prior investigation revealed that a bilateral intrahippocampal injection of Clobenpropit significantly ameliorated spatial memory impairments following MK801 treatment in rats. This improvement was attributed to its ability to modulate various neurotransmitters, including histamine, dopamine, acetylcholine, and serotonin [142]. In another study, Clobenpropit exhibited a protective role against mitochondrial dysfunction and neuroinflammation. The investigation assessed the effect of a 30-day pretreatment with Clobenpropit on cognitive impairment, mitochondrial dysfunction, and neuroinflammation induced by LPS. The outcomes of the study exhibited improved spatial learning and memory with the administration of Clobenpropit. Additionally, Clobenpropit exhibited anti-inflammatory properties, reducing the levels of COX-2 enzymes and proinflammatory cytokines while elevating anti-inflammatory cytokines in the brain. In conclusion, the results proposed that Clobenpropit holds promise as a neuroprotective agent for addressing neuroinflammation, cognitive decline, and mitochondrial damage in the brain [143]. 

Multiple studies have revealed that enhancing autophagy can have a positive effect on learning behavior and the reduction of Aβ accumulation and BACE1 degradation [144,145]. The outcomes of a study indicated that thioperamide improved cognitive deficits in mice with APP/PS1 mutations by upregulating the autophagy levels that were blocked by an autophagic inhibitor called 3-MA. The in vitro studies further confirmed that thioperamide exerted protective effects on primary neurons against Aβ-induced injury by enhancing autophagy that were reversed significantly by inhibiting autophagy using 3-MA or siRNA targeting Atg7. Furthermore, the study found that the reduced Aβ deposition and the accumulation of BACE1 in the hippocampus and cortex, both associated with Aβ pathology, were reversed by inhibiting autophagy using 3-MA. This suggests that the improved effect of thioperamide on Aβ pathology is linked to the upregulation of autophagy [138]. Consequently, the utilization of H3R antagonists and inverse agonists have demonstrated promise in decreasing the buildup of Aβ aggregates and promoting autophagy through the involvement of the CREB protein. Furthermore, these compounds have shown potential in ameliorating memory deficits. Also, by elevating histamine release and subsequently promoting hippocampal neurogenesis, these agents have been found to attenuate cognitive dysfunction.

#### 3.4.2. Parkinson’s Disease

PD is the second-most widespread neurodegenerative disease. PD is frequently associated with the degeneration of dopaminergic neurons in the substantia nigra pars compacta, resulting in dopamine depletion in the striatum [75]. The primary neurons projecting from the striatum are the GABAergic medium-spiny projection neurons (MSNs), which express either the D1 receptor (D1R) or the D2 receptor (D2R). Dopamine regulates the upper motor neurons through direct and indirect pathways. In the direct pathway, D1R-expressing MSNs extend projections to the substantia nigra pars reticulata and internal segments of the globus pallidus, promoting motor activation. In the indirect pathway, D2R-expressing MSNs send projections through the external part of the globus pallidus [1]. In PD, there is reduced activity in the D1R-positive direct pathway MSNs and increased activity in the D2R-positive indirect pathway MSNs. This imbalance results in the heightened firing of GABAergic nigrothalamic neurons and diminished activity in thalamocortical pathways [96]. Approximately 85% of MSNs expressing both D1R and D2R in the striatum also contain H3R, which can potentiate D2R effects and inhibit D1R effects [146]. H3R interact differentially with D1R and indirect D2R MSNs in the striatum. H3R and D2R potentiate each other, while H3R inhibits D1R effects. Overall, H3R has complex, opposing interactions with D1R and cooperative interactions with D2R in striatal regulation [97]. 

The important role of microglia in the neuroinflammatory response in PD has been documented [147]. Consequently, targeting the suppression of microglial activation can be a potential therapeutic strategy in the treatment of PD [148]. There have been inconsistent findings regarding the involvement of the neuronal histaminergic system. Post-mortem analysis of brain tissues from individuals with PD has revealed a significant accretion of Lewy bodies or Lewy neurites in the TMN, indicating its severe damage during the progression of PD. A study reported elevated histamine levels in specific regions responsible for motor behavior, such as the substantia nigra, putamen, and globus pallidus, in PD patients [149]. The elevated density of histaminergic fibers has been found in the brains of patients with PD. Although histamine production in the TMN remains relatively unchanged in PD, the altered expression of histamine-related genes in regions receiving innervation from histaminergic neurons could potentially contribute to PD pathology [150,151]. Several investigations have provided evidence of the amelioration of various symptoms in PD through the use of H3R antagonists [152]. In post-mortem cases of PD, an increase in histaminergic nerve fibers, along with an upregulation of H3R, was observed [153]. This study revealed that the prolonged administration of H3R ligands, such as Clobenpropit, led to a notable decrease in brain pathology. This reduction was accomplished through the lowering of phosphorylated τ-protein and α-synuclein levels in the cerebrospinal fluid. Furthermore, Masini et al. (2017) showed that thioperamide rescued the normal rest/activity cycle and attenuated memory impairment in an experimental model of parkinsonism [154]. Targeting the histaminergic system in PD is an area of interest worth exploring [155]. In the therapy of PD, the deactivation of MAO A or MAO B has been established as a fundamental principle. Furthermore, a high expression of MAOs in neuronal tissues is believed to contribute to oxidative stress, leading to increased neuronal cell death. The H3R antagonist Ciproxifan has showed efficacy on both enzyme isoforms [156]. In a single-blinded trial involving patients with PD, Pitolisant has been found to alleviate excessive sleepiness without significant effects on the motor performance. Hence, the compounds that enhance local histamine release in specific brain regions may offer therapeutic insights for the treatment of PD.

#### 3.4.3. Autism Spectrum Disorder

Multiple lines of evidence indicate the beneficial use of H3R antagonists in reducing autism-like stereotypes and repetitive behaviors in different models for ASD [29,157,158,159]. Considering the growing body of research, previous studies have reported indications of ongoing neuroinflammatory processes in different brain regions, which encompass microglial activation, among individuals with ASD and other neuropsychiatric conditions [88]. ASD has been associated with the diagnosis of chronic or excessive neuroinflammation. The persistent activation of glia and changes in inflammatory function observed in this context may contribute to the behavioral characteristics seen in ASD [91,160]. The prolonged microglial activation due to the excessive release of proinflammatory cytokines is associated with neuronal cell death and the loss of synaptic connections leading to detrimental processes of synaptic dysfunction and neurodegeneration, ultimately contributing to the pathology of various neurological conditions [161,162]. Furthermore, previous research findings have highlighted the significant involvement of NF-κB in neuroinflammation. Research has indicated a significant increase in NF-κB expression within activated microglial cells in both experimental animals and post-mortem samples from individuals diagnosed with ASD [163]. These observations underscore the potential contribution of NF-κB in driving neuroinflammatory processes associated with neurological and neuropsychiatric disorders. This association emphasizes the relevance of targeting neuroinflammatory mechanisms in the development of appropriate therapeutic strategies. 

Interestingly, numerous histamine H3R antagonists exhibited promising results in various neurological disorders by targeting neuroinflammation such as H3R antagonist ST713, which decreased NF-κB and considerably reduced the elevated levels of measured cytokines in BTBR mice, a strain of mice with idiopathic autistic features [164]. A study conducted by Eissa et al. in 2019 revealed that TNF-α, IL-1, and IL-6 were elevated in autistic mice [165]. These findings align with previous research indicating that heightened neuroinflammation in individuals with autism is associated with elevated levels of TNF-α, IL-1, IL-6, and TGF-β [161,166]. Furthermore, when E100, a dual-active H3 receptor antagonist and acetylcholinesterase inhibitor, was systemically administered to valproic acid-exposed mice, it greatly reduced the levels of proinflammatory cytokines. Additionally, the neuroprotective effects of E100 against heightened levels of proinflammatory cytokines were reversed when combined with a CNS penetrant H3R agonist, underscoring the involvement of brain histamine in the neuroprotection shown by E100 in mice exhibiting ASD-like traits. The delivery of E100 significantly lowered the heightened levels of NF-κB. These findings indicate that, in animals with ASD-like characteristics, brain histamine plays a pivotal role in enhancing the therapeutic efficacy of E100. Further, immunofluorescence labeling showed that, when mice were exposed to valproic acid, they exhibited a higher presence of activated microglia. However, when valproic acid-exposed mice were treated with E100, microglial activation significantly decreased, indicating a reduction in microglial activation due to E100. Importantly, this effect caused by E100 was completely reversed upon the co-administration of (*R*)-α-methyl histamine, an agonist at H3R, suggesting that brain histamine plays a role in facilitating E100’s protective effects against disturbed microglia in these mice [165]. 

In another investigation, it was observed that exposure to valproic acid significantly increased the expression of proinflammatory cytokines (IL-1, IL-6, and TNF-α) in mice when subjected to LPS. However, treatment with DL77, an antagonist at H3R, markedly reduced these proinflammatory cytokines [158]. An additional noteworthy observation from the study was the abrogation of the protective effects of DL77 on proinflammatory cytokines upon administration of the H3R agonist, which further suggests the involvement of histaminergic neurotransmission in facilitating the neuroprotective function of DL 77. Neuroinflammation plays a pivotal role in the behavioral manifestations observed in ASD [167]. Therefore, a potential therapeutic approach for treating ASD may involve the mitigation of neuroinflammation by managing microglial activation and the suppression of proinflammatory mediators and free radicals. The role of histamine in targeting microglial activation and suppressing proinflammatory neurotoxicity could represent an effective therapeutic strategy for fostering neuroprotection and mitigating ASD-like behaviors. The current evidence indicates a potential association between chronic neuroinflammation and cognitive deficits, with preclinical studies demonstrating the ameliorative effects of H3R antagonists and inverse agonists on neuroinflammatory processes in ASD.

### 3.5. Procognitive Effects of H3R Modulators in Preclinical Studies 

The literature consistently highlights the beneficial effects of various H3R antagonists and inverse agonists in addressing cognitive damage in AD, PD, anxiety, schizophrenia, depression, and sleep disorders [4,99,168,169,170]. These findings provide a promising approach for addressing neuropathological features that commonly cooccur. The treatment with (*R*)-α-methylhistamine (RAMH), a CNS-penetrant H3R agonist, has been demonstrated to negatively impact cognitive function in various preclinical models [4]. Conversely, studies involving H3R knockout mice have shown enhanced cognitive function [171]. Furthermore, cognitive deficits have been alleviated through the inhibition of H3R by antagonists, as observed in several rodent models [172,173,174]. Manipulating the histaminergic system through either pharmacological means with α-FMH or genetically with HDC-/- did not lead to any impairment in short-term recognition memory or fear memory within the inhibitory avoidance paradigm in mice [175]. The H3R agonist VUF16839 hindered short-term social recognition tests in mice, and this impairment was reversed by donepezil, indicating the activation of H3-heteroreceptors regulating acetylcholine release rather than histamine [175]. H3R antagonists/inverse agonists show potential therapeutic benefit in enhancing spatial memory. 

In a study using the Morris water maze, for evaluating spatial-working memory S38093, a H3R antagonist/inverse agonist demonstrated an improvement in scopolamine-induced amnesia [176]. Similarly, improved escape latencies in the Morris water maze following the administration of the GSK189254 H3R antagonist/inverse agonist was observed in a rodent model of amnesia [172]. In preclinical models, CUS was observed to decrease the BDNF levels in both the prefrontal cortex and hippocampus of mice, which was countered by the H3R antagonist ciproxifan [177]. Another H3R antagonist, JNJ-10181457, demonstrated antidepressant-like properties in a depression model induced by LPS. It also reduced the immobility time in TST and the generation of proinflammatory cytokines from microglial cells [123]. ST-1283, a H3R antagonist, reduced depression-like behaviors evaluated in FST, TST, and a novelty suppressed feeding test [178]. Table 4 and Table 5 summarize various preclinical and clinical studies of H3R modulators in various brain disorders.

While numerous preclinical studies have demonstrated the potential of various H3R antagonists and inverse agonists to enhance the cognitive abilities in rodents, clinical trials involving agents such as GSK239512 and ABT-288 have yielded limited efficacy in improving cognitive functions among patients with mild-to-moderate AD. While GSK239512 demonstrated some improvement in episodic memory, it did not affect working memory. Overall, H3R antagonists had modest and selective effects on cognitive function based on clinical observations [211,212].

## 4. Discussion

In recent times, notable attention has been directed towards the design and development of H3R antagonists as a potential therapeutic strategy for CNS-related disorders. Mounting evidence indicates a substantial involvement of neuroinflammation in the initiation and progression of cognitive decline in various neurological disorders [213,214]. The targeting of neuroinflammatory processes associated with various neurodegenerative diseases has emerged as a promising therapeutic approach. Extensive preclinical research has provided compelling evidence supporting the potential application of H3R antagonists in addressing cognitive impairments associated with AD and other related conditions [104]. In 1986, De Almeida and Izquierdo proposed the role of histamine in brains regulating different memory phases [215]. Extensive animal studies have provided substantial evidence regarding the cognition-promoting benefits of thioperamide and other H3R inverse agonists/antagonists [193,197,203,216]. 

While histamine is recognized for its role in enhancing inflammation in the peripheral system, there is growing evidence suggesting a dual role for histamine in modulating microglial inflammatory responses within the CNS 2]. Histamine itself can activate microglia and promote inflammation, but it also demonstrates anti-inflammatory effects under conditions of stress-induced pathology [111]. Additionally, H1R activation leads to microglial activation and proinflammatory effects, whereas activation of the H2R leads to microglial inhibition and anti-inflammatory effects [108,110]. H3R ligands with neuroprotective properties have the potential to prevent neuronal degeneration and slow down the progression of various brain disorders [71]. Most of the recent research work has demonstrated that there is a connection between neuroinflammation and neurodegenerative disorders, and H3 receptor antagonists demonstrated a favorable impact by reducing the inflammatory biomarkers, suggesting the potential role of these antagonists to concurrently regulate vital brain neurotransmissions and influence numerous signaling pathways related to the development of AD and PD, such as the PI3K/AKT/GSK-3β pathway [217]. 

In individuals with AD, several histopathological changes are observed, including impaired cholinergic neurotransmission, Aβ plaque buildup within the brain, the presence of intracellular NFT’s, and the advancement of oxidative stress and inflammation-induced damage. The current drugs approved for AD offer only symptomatic relief but fail to halt neurodegeneration. Given the complexity of the disease, relying on a single compound targeting a specific receptor is insufficient to achieve significant therapeutic effects [218]. To address this challenge, a new pharmacological approach involves designing single molecules capable of simultaneously modulating multiple targets [170,219]. These molecules are known as multitarget-directed ligands (MTDLs) and are primarily developed as potential therapeutics for treating multifactorial diseases like neurodegenerative disorders [220]. There has been significant interest in developing multitarget compounds that include H3R-blocking properties [221]. Eissa et al. (2019) assessed the impact of a novel dual-active ligand, E100, on autism-like behaviors and neuroinflammation in an animal model of ASD developed by valproic acid exposure. In this study, E100, which has a high affinity for H3R antagonism and balanced acetylcholinesterase inhibition, showed dose-dependent improvement in repetitive and compulsive behaviors. E100 also attenuated anxiety levels in the mice. Additionally, pretreatment with E100 led to a decrease in microglial activation. The proinflammatory cytokines and the expression of iNOS, COX-2, and NF-κB were also reduced in the cerebellum and hippocampus [165]. 

In another study systemic treatment with a novel antagonist targeting H3R/D2R/D3R receptors, called ST-2223, improved social deficits and reduced anxiety levels associated with ASD in male Black and Tan Brachyury (BTBR) mice [209,210]. Furthermore, the study demonstrated its beneficial effects in reducing oxidative stress, as evident by a decrease in brain malondialdehyde levels and elevation in the levels of reduced glutathione, superoxide dismutase, and catalase. Patnaik et al. (2018) demonstrated a noteworthy decrease in the breakdown of the blood–brain barrier, deposition of the Aβ peptide, and neuronal damage with the treatment with BF-2649, a H3R inverse agonist, and Clobenpropit, a H3R antagonist with partial H4 agonist properties. Clobenpropit demonstrates a more effective neuroprotective capacity compared to BF-2649 when administered at equivalent doses [141]. The compounds exhibiting H3 antagonistic action along with partial H4 agonistic activity could provide increased therapeutic potential for mitigating pathologies linked to AD. Studies in mice have demonstrated that H3R antagonists/inverse agonists can induce hippocampal neurogenesis, offering potential advantages for addressing age-related cognitive deficits [6]. 

Ghamari et al. (2019) reviewed the challenges in designing non-imidazole-based H3R antagonists/inverse agonists. Obstacles include an affinity for hERG K^+^ channels causing cardiotoxicity, phospholipidosis concerns, and prolonged duration of action, leading to insomnia. Receptor occupancy over 80% induces insomnia, emphasizing the importance of appropriate dosage schemes in clinical investigations. Characterizing ligands with brief-to-moderate receptor residence times is suggested to address this issue. The complex pharmacology of H3R (splice variants, oligomerization, constitutive activity, and differential signaling pathways) complicates the design process. The authors recommend special attention to the structural information for a rational compound design and using assays with multiple H3R isoforms for a comprehensive assessment [98].

Overall, the inhibition of H3R to regulate histamine release can offer numerous advantageous outcomes for brain health. It can reduce the activity of microglial cells, promote a transition from a proinflammatory state (M1) to an anti-inflammatory state (M2), mitigate neuroinflammation, and enhance hippocampal neurogenesis. As a result, this intervention can ultimately enhance cognitive function and has potential therapeutic outcomes. Considering the preclinical outcomes, targeting brain H3R with novel H3R antagonists represents a promising avenue, holding potential implications for the treatment of neurocognitive conditions.

## 5. Conclusions and Future Perspectives

Advancements in understanding neurotransmitter systems and synaptic neurophysiology have facilitated progress in the management of neuropsychiatric and neurodegenerative disorders. Microglia influence the inflammatory response that occurs in various pathologies, contributing to neuroinflammation. Accumulating evidence indicates a potential association between cognitive deficits and chronic neuroinflammation. Numerous lines of evidence show the significant contribution of the histaminergic system in the regulation of neurological disorders. Histamine H3R antagonists/inverse agonists are a promising and novel category of medications that have demonstrated the ability to alleviate various neuroinflammatory processes and improve cognitive function in preclinical models of AD, PD, epilepsy, depression, and autism. Recognizing microglia’s pivotal role in neuroinflammation and neurodevelopment and modulating microglial activation through H3R antagonists present a potential therapeutic strategy in neurological disorders exhibiting neuroinflammation. H3R antagonists/inverse agonists can serve as active elements in dual- or multitargeting drugs designed for the treatment of neurocognitive disorders. Advances in chemo-genetic approaches can provide more insights into these neurological disorders, aiding in the development of therapeutics to target the histaminergic system. Further investigations are needed to validate the involvement of different signaling pathways in the beneficial effects of H3R antagonists in animal models of AD, PD, and ASD. Additionally, there is a need to assess the precise impact of H3R antagonists on diverse neurotransmitters within the brain, elucidating their role in the observed neuroprotective effects. Furthermore, it is crucial to explore the specific functions of histamine receptors in distinct regions of the brain and cell types, which would facilitate the development of targeted and specific ligands for histamine receptors, ultimately leading to more efficient treatment options.

## Figures and Tables

**Table 1:** Promising medications targeting neuroinflammation in AD.

Compound	Mechanism of Action	NCT Identifier
Indomethacin	COX1/2 inhibitor	NCT00432081
Simufilam	Inhibitor of Filamin A protein; reducing Aβ levels and mitigating synaptic dysfunction	NCT05575076
Candesartan	Decreases NO and TNF-α levels	NCT02646982
Minocycline	Inhibition of eIF2α	NCT01463384
Pioglitazone	PPARγ agonist	NCT00982202
Simvastatin	immuno-modulatory and anti-inflammatory	NCT00486044
Atomoxetine	Reduces NF-κB expression	NCT01522404
Baricitinib	Janus kinase inhibitor; reduces neuroinflammation	NCT05189106
AL002	Monoclonal antibody, enhance microglial clearance of Aβ	NCT04592874
NE3107	inhibits activation of NF-κB; MAPK-1/3 inhibitor	NCT04669028
Nilvadipine	Lower brain amyloid and improve memory function	NCT02017340.
Masitinib	tyrosine kinase inhibitor	NCT01872598.
Baricitinib	Janus kinase inhibitor; reduces neuroinflammation	NCT05189106
L-Serine	Dietary amino acid, reduces inflammation in brain	NCT03062449
Montelukast	Acts as an antagonist at the Cysteinyl leukotriene type 1 receptor, reducing buildup of Aβ protein.	NCT03402503
Pepinemab (VX15)	monoclonal antibody targeting semaphorin 4D, alleviates inflammation.	NCT04381468
Senicapoc	Calcium-activated potassium channel blocker	NCT04804241
Canakinumab	Anti-IL-1β monoclonal antibody	NCT04795466
Edonerpic (T-817MA)	stimulates sigma receptors, safeguard synaptic plasticity and offers defense against Aβ aggregates.	NCT04191486
Neflamapimod (VX-745)	p38 MAPK-α inhibitor; reduces synaptic dysfunction	NCT03435861
Edicotinib (JNJ-40346527)	reduces neurodegeneration and microglial proliferation	NCT04121208
Emtricitabine	reduces neuroinflammation.	NCT04500847
XPro1595	TNF inhibitor; reduces neuroinflammation	NCT03943264
Celecoxib	COX-2 inhibitor	NCT00065169
Entanercept	TNF-α inhibitor	NCT00203359
Cyclophosphamate	Immuno-suppressor and alkylating agent	NCT00013650

**Table 2:** Promising medications targeting neuroinflammation in PD.

Compound	Mechanism of Action	NCT Identifier
Semaglutide	anti-inflammatory and neuroprotective in idiopathic PD	NCT03659682
Rifaximin	Modifies Gut Microbiota and Attenuates Inflammation	NCT03958708
Sargramostim	Recombinant GM-CSF	NCT01882010
Exenatide	GLP1 analogue	NCT01971242
Pioglitazone	PPARγ agonist	NCT01280123
Nabilone	Cannabinoid system agonist	NCT03769896
PRX002	mAb directed at α-synuclein	NCT02157714
BIIB054	mAb targeting α-synuclein	NCT02459886
AFFITOPE PD01A	vaccine targeting α-synuclein	NCT02216188
AFFITOPE PD03A	vaccine targeting α-synuclein	NCT02267434
UB-312	vaccine targeting α-synuclein	NCT04075318
Radotinib	c-Abl Tyrosine kinase inhibitor	NCT04691661
K0706	c-Abl Tyrosine kinase inhibitor	NCT03655236
FB-101	c-Abl Tyrosine kinase inhibitor	NCT04165837
Sulforaphane	Anti-inflammation and neuroprotection	NCT05084365
Montelukast	cysteinyl LT 1 antagonist	NCT06113640

**Table 3:** Promising medications in clinical trials for ASD.

Compound	Mechanism of Action	NCT Identifier/Reference
Taurine	Amino acid	NCT05980520
Prednisolone	Corticosteroid	[95]
L1-79	Tyrosine hydroxylase inhibitor	NCT05067582
STP1	PDE inhibitor and an NKCC1 inhibitor	NCT04644003
Minocycline	Modulates microglia polarization and neuroinflammation	NCT04075318
N-acetylcysteine	Antioxidant	NCT03008889
Sulforaphane	Anti-inflammation and neuroprotection	NCT02879110
Tideglusib	GSK-3 inhibitor.	NCT02586935
Oxytocin	Regulation of synaptic function	NCT01944046
Luteolin	antioxidant, anti-inflammatory and neuroprotective effects.	NCT01847521
Omega-3 Fatty Acids	Lower inflammation in brain	NCT01695200

**Table 4:** Summary of the preclinical studies of H3R modulators in various neurological disorders.

H3R Modulators	Pharmacological Effect	Reference
**H3R Antagonists**		
DL77	Reduced stereotypies and social deficits in preclinical investigations in a model of ASD, attenuated the increase in TNF-α, IL-6, and IL-1β.	[158]
	Improved cognitive impairments in dizocilpine induced memory impairment in rats.	[179]
	Increased anticonvulsant activity in epilepsy models.	[180]
	Dose dependent reduction in both ethanol intake and preference.	[181]
E177	Enhancement of memory in PTZ-kindled animals, mitigation of oxidative stress.	[182]
E169	Improvement of memory impairments caused by MK-801.	[183]
ST-1283	Anxiolytic and antidepressant-like effect	[178]
Ciproxifan	Reduction in hyperactivity, and memory impairment in APP_Tg2576_ mice.	[184]
	Attenuation of impaired sociability and repetitive behavior in the VPA model of ASD.	[157]
	Improved retrieval of contextual memory in stress and nonstress conditions.	[185]
	Alleviation of learning and memory decline in ASD model induced by VPA.	[186,187]
	Improvement in depression-like behavior.	[177]
JNJ10181457	Restored cognitive function following scopolamine-induced deficits in rats. Normalized Acetylcholine neurotransmission in a model of cognitive impairment.	[173]
ABT-239	Reduced Ketamine/MK-801 induced impairments in cognitive tests in rodents.	[188]
	Attenuated KA-mediated behavioral anomalies and excitotoxicity.	[189]
	Improved attention and cognition, as demonstrated by improved performance in inhibitory avoidance tests and social memory tasks.	[190]
	Prevented cognitive deficits following chronic restraint stress.	[191]
ABT-288	Improvement in social recognition memory, spatial learning, and reference memory.	[192]
GSK189254	Improved cognitive performance in object recognition, passive avoidance, and water maze tests.	[172,193]
Thioperamide	Enhanced pre-pulse inhibition in schizophrenia model.	[194]
	Reversed amnesia caused by scopolamine and dizocilpine.	[195]
	Improved cognitive function in a model of cognitive impairment induced by scopolamine.	[196]
	Rescues the normal rest/activity cycle and improved memory function in experimental parkinsonism.	[154]
SAR110894	Prevented tau aggregation in the hippocampus. NFTs were reduced in the hippocampus, and amygdala, mitigation of episodic memory deficits.	[174,197]
SAR110068	Attenuated memory and attentional deficits	[197]
	Decreased slow-wave sleep	[198]
Enerisant	Mitigated the decline in object and social memory induced by scopolamine.	[199]
CEP-26401 (irdabisant)	Enhanced performance in the rat social recognition model assessing short-term memory.	[200]
ST713	Attenuated expression of NF-kB and considerably reduced elevated levels of the measured cytokines in BTBR mice, mitigated repetitive self-grooming and aggression.	[29]
	Dose-dependent improvement of social deficits, alleviation of repetitive behaviors in model of ASD. Reduced levels of NF-κB p65, COX-2, and iNOS.	[164]
Clobenpropit	Marked improvement in the deficits of working and reference memory induced by MK-801.	[142]
	Reversed memory deficits, No effects on anxiety-like behavior.	[201]
	Reduction in behavioral deficits. Lowered proinflammatory cytokines and elevated levels of anti-inflammatory cytokines in brain tissues.	[143]
	Attenuated memory impairment induced by scopolamine.	[202]
**H3R inverse agonists**		
BF 2649	Significant decreases in injuries to neuronal and glial cells, decrease in the number of Aβ positive cells in the cortex in a model of AD in rats.	[141]
	Improves fear memory and reverses memory deficits induced by dizocilpine.	[203]
S 38093	Improved spatial working memory, improvement in cognition.	[176]
Samelisant (SUVN-G3031)	Enhanced cognitive function in social recognition and object recognition task	[204]
**Dual-active H3R antagonists and AChE inhibitors (AChEI)**		
UW-MD-71	Improved performance and enhanced memory. Ameliorated the dizocilpine-induced amnesic effects.	[205]
UW-MD-72	Reduction in memory impairments caused by Scopolamine and dizocilpine.	[206]
E100	Reduced repetitive stereotypic behaviors in autistic mice, mitigated oxidative stress with decreased microglial activation and decreased levels of NF-κB, COX-2, and iNOS.	[165,207,208]
**Multiple-active H3R/D2R/D3R antagonists**		
ST-2223	Improvement in core ASD-related behaviors and modulated altered levels of monoaminergic neurotransmitters, mitigation of oxidative stress.	[209,210]

**Table 5:** Summary of clinical trials involving H3R antagonists and inverse agonists.

Compound	Clinical Indication/Condition	Clinical Trial Identifier	Status
GSK239512	Mild-to-moderate AD	NCT00675090	Completed
GSK239512	Schizophrenia	NCT01009060	Completed
ABT-288	Mild-to-moderate AD	NCT01018875	Completed
AZD5213	Mild-to-moderate AD	NCT01548287	Completed
AZD5213	Tourette’s Disorder	NCT01904773	Completed
GSK189254	Mild cognitive impairment, dementia	NCT00474513	Completed
SAR110894	Add-on therapy for mild-to-moderate AD	NCT01266525	Completed
TS-091	Narcolepsy	NCT03267303	Completed
JNJ-17216498	Narcolepsy	NCT00424931	Completed
BF2.649	Narcolepsy	NCT01638403	Completed
PF-03654746	Narcolepsy	NCT01006122	Completed
GSK239512	Schizophrenia	NCT01009060	Completed
MK0249	Schizophrenia	NCT00506077	Completed
ABT-288	Schizophrenia	NCT01077700	Completed
GSK239512	AD	NCT01009255	Completed
MK0249	AD	NCT00420420	Completed
ABT-288	AD	NCT01018875	Completed
BF2.649	PD	NCT01036139	Completed
		NCT01066442	Completed
JNJ-31001074	Attention-Deficit/Hyperactivity Disorder (Adults)	NCT00566449	Completed
MK-3134	Dementia	NCT01181310	Completed
BF2.649	EDS in Obstructive sleep apnea	NCT02739568	Completed
BF2.649	Autism Spectrum Disorder	NCT05953389	Not yet recruiting
GSK189254	Hyperalgesia	NCT00387413	Completed

## Data Availability

This manuscript is a review, and most of the studies referred to herein this article have been appropriately cited in the manuscript.

## Abbreviations

AChEI	Acetylcholinesterase inhibitors
Aβ	Amyloid-beta
ALR	Absent in melanoma 2 (AIM2)-like receptor
AKT	Protein kinase B
BACE1	β-site amyloid precursor protein cleaving enzyme 1
BDNF	Brain-derived neurotrophic factor
BTBR	Black and Tan Brachyury
CBP	CREB Binding Protein
CLR	C-type lectin receptor
CREB	cAMP-responsive element binding protein
CSF 1	Colony stimulating factor-1 (CSF-1)
CUS	Chronic unpredictable stress
DAMPs	Damage associated molecular pattern
EAE	Experimental autoimmune encephalomyelitis
FGF	Fibroblast growth factor
FST	Forced swimming test
GPCRs	G protein-coupled receptors
GABA	Gamma-aminobutyric acid
GDNF	Glial cell-derived neurotrophic factor
GSK3β	Glycogen synthase kinase 3β
HNMT	Histamine N-methyltransferase
IRF8	Interferon regulatory factor 8
LTP	Long-term potentiation
MAPKs	Mitogen-activated protein kinases
NADPH	Nicotinamide adenine dinucleotide phosphate
NFTs	Neurofibrillary tau tangles
NF-κB	Nuclear factor kappa light chain enhancer of activated B cells
NLR	Nod-like receptors
NGF	Nerve growth factor
PAMPs	Pathogen associated molecular pattern
PI3K	Phosphatidylinositol 3-kinase
RAMH	(*R*)-α-methylhistamine
ROS	Reactive Oxygen Species
RLR	Retinoic acid-inducible gene-I (RIG-I)-like receptor
TLR	Toll-like receptor
TMN	Tuberomammillary nucleus
TNFα	Tumor Necrosis Factor α
TGFβ	Transforming growth factor β
TST	Tail suspension Test
VEGF	Vascular endothelial growth factor
